# Cellulases from Thermophilic Fungi: Recent Insights and Biotechnological Potential

**DOI:** 10.4061/2011/308730

**Published:** 2011-11-17

**Authors:** Duo-Chuan Li, An-Na Li, Anastassios C. Papageorgiou

**Affiliations:** ^1^Department of Environmental Biology, Shandong Agricultural University, Taian, Shandong 271018, China; ^2^Turku Centre for Biotechnology, University of Turku and Åbo Akademi University, 20521 Turku, Finland

## Abstract

Thermophilic fungal cellulases are promising enzymes in protein engineering efforts aimed at optimizing industrial processes, such as biomass degradation and biofuel production. The cloning and expression in recent years of new cellulase genes from thermophilic fungi have led to a better understanding of cellulose degradation in these species. Moreover, crystal structures of thermophilic fungal cellulases are now available, providing insights into their function and stability. The present paper is focused on recent progress in cloning, expression, regulation, and structure of thermophilic fungal cellulases and the current research efforts to improve their properties for better use in biotechnological applications.

## 1. Introduction

Cellulose is one of the main components of plant cell wall material and is the most abundant and renewable nonfossil carbon source on Earth. Degradation of cellulose to its constituent monosaccharides has attracted considerable attention for the production of food and biofuels [1, 2]. The degradation of cellulose to glucose is achieved by the cooperative action of endocellulases (EC 3.1.1.4), exocellulases (cellobiohydrolases, CBH, EC 3.2.1.91; glucanohydrolases, EC 3.2.1.74), and beta-glucosidases (EC 3.2.1.21). Endocellulases hydrolyze internal glycosidic linkages in a random fashion, which results in a rapid decrease in polymer length and a gradual increase in the reducing sugar concentration. Exocellulases hydrolyze cellulose chains by removing mainly cellobiose either from the reducing or the non-reducing ends, which leads to a rapid release of reducing sugars but little change in polymer length. Endocellulases and exocellulases act synergistically on cellulose to produce cellooligosaccharides and cellobiose, which are then cleaved by beta-glucosidase to glucose [3].

Thermophilic fungi are species that grow at a maximum temperature of 50°C or above, and a minimum of 20°C or above [4]. Based on their habitat, thermophilic fungi have received significant attention in recent years as a source of new thermostable enzymes for use in many biotechnological applications, including biomass degradation. Thermophilic cellulases are key enzymes for efficient biomass degradation. Their importance stems from the fact that cellulose swells at higher temperatures, thereby becoming easier to break down. A number of thermophilic fungi have been isolated in recent years and the cellulases produced by these eukaryotic microorganisms have been purified and characterized at both structural and functional level. This review aims at presenting up-to-date information on molecular, structural, genetic, and engineering aspects of thermophilic fungal cellulases and to highlight their potential in biotechnological applications.

## 2. Cloning, Expression and Regulation of Cellulase Genes from Thermophilic Fungi

### 2.1. Regulation of Gene Expression

Production of fungal cellulases is commonly induced mainly in the presence of cellulose and is controlled by a repressor/inducer system [5]. In this system, cellulose or other oligosaccharide products of cellulose degradation act as inducers while glucose or other easily metabolized carbon sources act as repressors [6–10]. It has been demonstrated that the upstream regulatory sequence (URS) in fungal cellulase gene promoters plays a key role in the regulation of glucose repression [11, 12]. In *Trichoderma reesei*, the protein product of the regulatory gene *cre1* (a Cys_2_His_2_ zinc finger protein) is a negatively acting transcription factor that binds to DNA consensus sequence SYGGRG (where S = C or G, Y = C or T, R = A or G) in the URS and represses transcription of cellulase genes in the presence of glucose [11]. In addition, three new transcription factors (ACEI, ACEII, and XYR1) have been identified in *T. reesei* and implicated in cellulase gene regulation [12]. Thermophilic fungal cellulases have also been found to possess a repressor/inducer system [4]. Unlike the transcription factors involved in *T*.* reesei* cellulase gene regulation, the full repertoire of transcription factors influencing cellulase gene expression in thermophilic fungi has not been described to date. Nevertheless, potential regulatory element consensus sequences have been identified in the 5′ upstream region of thermophilic fungal cellulase genes (6, 9, 13–15), and CREI genes from two thermophilic fungi (*Talaromyces emersonii* and *Thermoascus aurantiacus*) have been cloned (GenBank AF440004 and AY604200, resp.). It is, therefore, likely that cellulase gene regulation in thermophilic fungi may share certain similarities with *T*.* reesei*.

In a similar fashion as in mesophilic fungi, multiple forms of cellulases are also produced in thermophilic fungi [4]. *Humicola grisea*, for example, has four cellobiohydrolases in family 7 while *Aspergillus niger* (a mesophilic fungus) two. The observed multiplicity of cellulolytic enzymes may be the result of genetic redundancy [13, 14] or the outcome of differential posttranslational and/or postsecretion processing [4].

### 2.2. Heterologous Expression

About 50 genes encoding thermophilic fungal cellulases have been isolated, analyzed, and expressed. A brief summary is given in Table 1. Cellulases are glycosyl hydrolases classified into families 1, 3, 5, 6, 7, 8, 9, 10, 12, 16, 44, 45, 48, 51, and 61 (http://www.cazy.org/). Thermophilic fungal cellulases are found in families 1, 3, 5, 6, 7, 12, and 45.

Most cloned cellulase genes of thermophilic fungi are expressed well in host organisms, such as *E*. *coli*, yeast, and filamentous fungi. Expression of some thermophilic fungal cellulase genes in heterologous hosts is summarized in Table 1. Transformation of* T. reesei* with two endochitinase genes from *Melanocarpus albomyces* resulted in an increase in cellulase activity several times higher than that of the parental *M. albomyces* strain [23]. The majority of the recombinant cellulases expressed in yeast and filamentous fungi are glycosylated [16, 18]. Both the strain and culture conditions can affect the type and extent of the glycosylation [29]. Notably, when a gene encoding a beta-glucosidase of *T. emersonii* was cloned into *T. reesei*, the secreted recombinant enzyme contained 17 potential N-glycosylation sites in its functionally active form [24]. Importantly, the glycosylation of cellulases could contribute further to the improvement of their thermostability as it has been previously reported [30]. However, extensive glycosylation in recombinant enzymes could lead to reduced activity and increased non-productive binding on cellulose [29].

## 3. Purification and Characterization of New Cellulases from Thermophilic Fungi

Purified thermophilic fungal cellulases have been characterized in terms of their molecular weight, optimal pH, optimal temperature, thermostability, and glycosylation. Usually, thermophilic fungal cellulases are single polypeptides although it has been reported that some beta-glucosidases are dimeric [31]. The molecular weight of thermophilic fungal cellulases spans a wide range (30–250 kDa) with different carbohydrate contents (2–50%). Optimal pH and temperature are similar for the majority of the purified cellulases from thermophilic fungi. Thermophilic fungal cellulases are active in the pH range 4.0–7.0 and have a high temperature maximum at 50–80°C for activity (Table 1). In addition, they exhibit remarkable thermal stability and are stable at 60°C with longer half-lives at 70, 80, and 90°C than those from other fungi.

The structural characteristics underpinning the increased stability of thermophilic proteins have been studied more extensively in thermophilic bacteria and hyperthermophilic archaea [32, 33]. It should be noted, however, that a common set of determinants for protein thermostability has not been established so far and several contributors to protein thermostability have been proposed. A recent analysis suggested that an increase in ion pairs on the protein surface and a stronger hydrophobic interior are the major factors supporting increased thermostability in proteins [34]. Compared with thermophilic proteins from thermophilic bacteria and hyperthermophilic archaea, the understanding of the nature and mechanism of thermostability of proteins from thermophilic fungi is relatively poor. Hence, further characterization of amino acid residues related to thermostability is necessary for comprehensive understanding of their role in the thermostability of cellulases from thermophilic fungi.

## 4. Structure of Thermophilic Fungal Cellulases

### 4.1. Primary Structure

**Figure 1:**
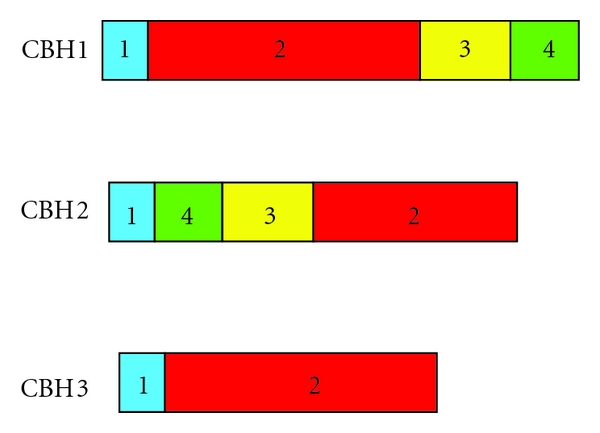
Domain organization of cellobiohydrolases CBH1 (AY861347), CBH2 (AY861348), and CBH3 of *C. thermophilum *(DQ085790) [16]. 1: signal peptide region, 2: catalytic domain, 3: hinge region, 4: cellulose-binding domain.

A common characteristic of cellulases is their modular structure. Typically, endocellulases and cellobiohydrolases are composed of four domains or regions (Figure 1): a signal peptide that mediates secretion, a cellulose-binding domain (CBD) for anchorage to the substrate, a hinge region (linker) rich in Ser, Thr and Pro residues, and a catalytic domain (CD) responsible for the hydrolysis of the substrate. The mature proteins are *O*- and *N*-glycosylated in the hinge region and the CDs, respectively. The effect of the glycosylation sites in the hinge region is not clear yet but they may play a role in the flexibility and disorder of the linker [35].

 Variations between cellulases within the same mechanistic class have been observed. An example is illustrated by *T. emersonii* CBHII, which is characterized by a modular structure [6] whereas CBH1 from the same fungus consists solely of a catalytic domain [7]. Similarly, *Chaetomium thermophilum* CBH1 and CBH2 consist of a typical CBD, a linker, and a catalytic domain. In contrast, CBH3 only comprises a catalytic domain and lacks a CBD and a hinge region [16].

Fungal CBDs are composed of less than 40 amino acid residues, and they interact with cellulose through a flat or platform-like hydrophobic binding site formed by three conserved aromatic residues. The binding site is thought to be complementary to the flat surfaces presented by cellulose crystals [36, 37]. The (110) faces of the cellulose crystalline microfibrils have been proposed as the putative CBD binding site [38]. With this arrangement, the glucopyranoside rings of cellulose are expected to be fully exposed and available for hydrophobic interactions.

Deletion of the CBDs from *T. reesei* Cel7A and Cel6A and *H. grisea* CBH1 greatly reduces enzymatic activity toward crystalline cellulose [48], suggesting that the tight binding to cellulose mediated by the CBD is necessary for the efficient hydrolysis of crystalline cellulose by these enzymes. Substitution of the three conserved aromatic residues (W494, W520, and, Y521) in *H. grisea* CBH1 CBD with other amino acids (G, F or W) has demonstrated the importance of these residues in the interdependency of high activity of *H. grisea* CBH1 on crystalline cellulose and high cellulose-binding ability [49].

### 4.2. Three-Dimensional (3D) Structure

**Figure 2:**
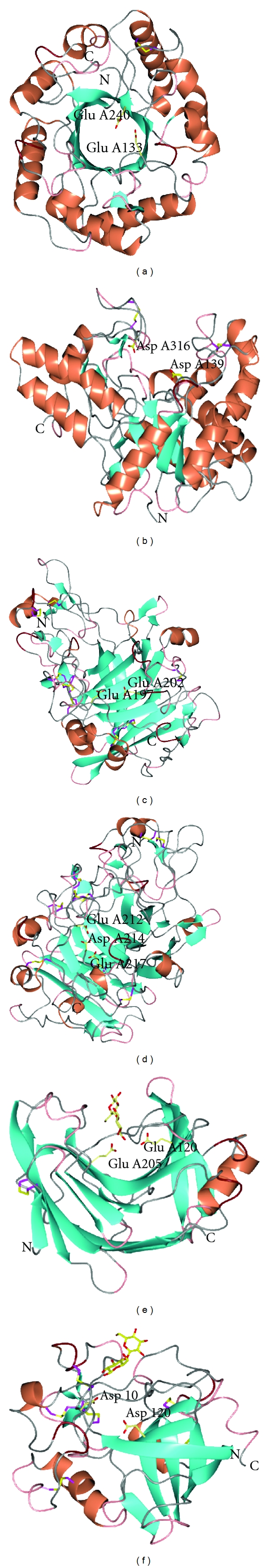
Ribbon diagrams of known thermophilic fungal cellulase structures. The catalytic residues are shown in stick representation. (a) *T. aurantiacus* family 5 endoglucanase (PDB id 1GZJ), F5 (b) *H. insolens* family 6 endoglucanase Cel6B (PDB id 1DYS), (c) *H. insolens* family 7 endoglucanase EGI (PDB id 2A39), (d) *M. albomyces* family 7 cellobiohydrolase in complex with cellotetraose (PDB id 2RG0), (e) catalytic domain of *H. grisea* family 12 Cel12A in complex with cellobiose (PDB id 1UU4), (f) *M. albomyces* family 45 endoglucanase in complex with cellobiose (PDB id 1OA7). *α*-Helices are shown in coral and *β*-strands in cyan. Bound ligands are depicted in stick representation and colored according to atom type. The figures of the structures were created with the CCP4 molecular graphics program [50].

Three-dimensional (3D) structures of thermophilic fungal cellulases from families 5, 6, 7, 12, and 45 have been reported (Table 2; Figure 2) and are briefly described below:

#### 4.2.1. Family 5

Family 5 cellulases belong to the endoglucanase type. The overall fold of the enzymes is a common *β*/*α*-barrel. In this family, only one structure from a thermophilic fungus, that of *T. aurantiacus* Cel5A, is known [45]. The structure consists solely of a catalytic domain. A substrate-binding cleft is visible at the C-terminal end of the barrel. The size and shape of the cleft suggest the binding of seven glucose residues (−4 to +3). In contrast to other family 5 cellulase structures, Cel5A has only a few extrabarrel features, including a short two-stranded *β*-sheet in *β*/*α*-loop 3 and three one-turn helices.

#### 4.2.2. Family 6

Family 6 comprises both endoglucanases and cellobiohydrolases. 3D structures have been reported for the endoglucanase Cel6B and the cellobiohydrolase Cel6A of this family from the thermophilic fungus *H. insolens* [39, 40]. The structures of these two cellulases exhibit a distorted *β*/*α*-barrel with the central *β*-barrel made up of seven instead of eight parallel *β*-strands. A substrate binding crevice is formed between strands I and VII. The crevice of Cel6A contains at least four substrate-binding sites, −2 to +2, whereas that of the Cel6B has six substrate-binding sites, −2 to +4. A significant difference between the endoglucanase Cel6B and the cellobiohydrolase Cel6A is that two extended surface loops enclose the active site in the Cel6A. These loops, however, are absent in Cel6B, resulting in an open substrate cleft in this endoglucanase. Because of this structural difference, endoglucanase can hydrolyze bonds internally in cellulose chains whilst cellobiohydrolase acts on chain ends.

#### 4.2.3. Family 7

Similarly to family 6, family 7 contains endoglucanases and cellobiohydrolases. Only a few structures of family 7 thermophilic fungal cellulases are currently known, including *T. emersonii* CBHIB [7], *H. insolens* EGI [41, 42], and *M. albomyces* Cel7B [47]. The structure of *M*.* albomyces* Cel7B, similar to *T. emersonii* CBHIB, is a representative of the family 7 cellobiohydrolases [7]. It consists of two antiparallel *β*-sheets packed face-to-face to form a *β*-sandwich. Both *β*-sheets contain six *β*-strands. Owing to their strong curvature, these two *β*-sheets form the concave and convex surfaces of the sandwich. The loops connecting the strands extend from the concave face of the sandwich and form an enclosed substrate-binding tunnel. The tunnel is about 50 Å long and contains nine substrate-binding sites, −7 to +2 [47]. 

*H. insolens* EGI has a *β*-sandwich structure similar to *M. albomyces* Cel7B (a cellobiohydrolase). The structure of EGI comprises two large antiparallel *β*-sheets consisting of seven and eight *β*-strands, respectively [41, 42]. However, there are structural differences between EGI and Cel7B. EGI, for instance, has an open long active site cleft in the center of a canyon formed by the curvature of the *β*-strands in the *β*-sandwich. In contrast, Cel7B has an enclosed substrate-binding tunnel [41, 47], which is similar to the endoglucanases and cellobiohydrolases of GH family 6. *C. thermophilum* CBH3 is a thermostable, single-module cellobiohydrolase with no 3D structure available [16]. This cellobiohydrolase shares high sequence identity (80%) with* M. albomyces* Cel7B. A homology model based on the *M*.* albomyces* Cel7B structure [47] showed that all the important residues in the catalytic site and substrate-binding site as well as the disulphide bonds present in *M. albomyces* Cel7B are also found in *C. thermophilum* CBH3.

#### 4.2.4. Family 12

The structure of a family 12 fungal cellulase from the thermophilic fungus *H. grisea* has been reported [44, 51]. It comprises 15 *β*-strands that fold into two antiparallel *β*-sheets, which pack on top of each other to form a compact curved *β*-sandwich. The convex *β*-sheet consists of six antiparallel strands, and the concave *β*-sheet consists of nine antiparallel strands. The structure's concave face creates a long substrate-binding cleft with six substrate-binding sites, −4 to +2.

#### 4.2.5. Family 45

The structures of two endoglucanases from family 45 have been solved: *H. insolens* Cel45A (EGV) [43] and *M. albomyces* 20 kDa endoglucanase [46, 52]. These two endoglucanases have a similar overall fold. Their structure consists of a six-stranded *β*-barrel with interconnecting loops. The molecule has the shape of a flattened sphere with approximate dimensions 32 Å × 32 Å × 22 Å. The *β*-strands are connected with long disulfide-bonded loop structures while the remainder of the structure is completed by three helices. A substrate-binding groove is formed between the *β*-barrel and the loop structures. This groove, approximately 40 Å long, 10 Å deep, and 12 Å wide, is subdivided into six substrate-binding sites, −4 to +2 [46].

## 5. Improvement of Thermophilic Fungal Cellulases

The current challenge in biomass conversion by cellulases concerns the degradation of cellulose in an efficient and cheap way. To increase cellulase efficiencies and to lower the cost, cellulases need to be improved to have higher catalytic efficiency on cellulose, higher stability at elevated temperatures and at nonphysiological pH, and higher tolerance to end-product inhibition [53]. Currently, two main research approaches used in the improvement of cellulases through protein engineering are: structure-based rational site-directed mutagenesis and random mutagenesis through directed evolution. Site-directed mutagenesis requires detailed knowledge of the protein's 3D structure. On the other hand, the directed evolution approach is not limited by the lack of the protein's 3D structure but requires an efficient method for high throughput screening [54].

### 5.1. Improvement of Thermostability

Although cellulases from thermophilic fungi are thermostable, the potential to increase their thermostability further would be beneficial for industrial applications. Improvement of *M. albomyces* Cel7B has been pursued by error-prone PCR, and 49 positive mutant clones were screened from 14600 random clones by a robotic high-throughput thermostability screening method [55]. Two positive thermostable mutants, Ala30Thr and Ser290Thr, showed improvements in unfolding temperatures (*T*_m_) by 1.5 and 3.5°C, respectively. In addition, the optimum temperature on a soluble substrate for the Ala30Thr mutant was improved by 5°C. The amino acid alterations are located in the *β*-strands furthest away from the active site tunnel of the Cel7B enzyme, which could improve protein packing. Recently, Cel7A cellobiohydrolase from the thermophilic fungus *T. emersonii* was engineered using rational mutagenesis to improve its thermostability and activity [25]. Additional disulphide bridges were introduced into the catalytic module of Cel7A. Three mutants had clearly improved thermostability as reflected by an improvement in Avicel hydrolysis efficiency at 75°C.

Structural analysis of *H. grisea* Cel12A, a thermostable endoglucanase, has revealed three unusual free cysteines in the enzyme: Cys175, Cys206, and Cys216. Subsequently, the following Cel12A mutants were constructed by site-directed mutagenesis: Cys175Gly, Cys206Pro, and Cys216Val. It was found that the three free cysteines play a significant role in modulating the stability of the enzyme [56]. More specifically, mutation of Cys206 to Pro and Cys216 to Val caused a reduction in the *T*_m_ of 9.1 and 5.5°C, respectively, compared to the wild-type enzyme. Moreover, when the free Cys175 was mutated to a Gly, the *T*_m_ of the enzyme was increased by 1.3°C. It has recently been reported that endoglucanases are characterized by variations in amino acid compositions resulting in fold-specific thermostability [57], thus providing new strategies for improvement of thermostability.

A new computational approach, SCHEMA, which uses protein structure data to generate new purpose-specific sequences that minimize structure disruption when they are recombined in chimeric proteins, has been employed to create thermostable fungal cellulases [21, 22]. The high resolution of *H. insolens* CBHII [39] as a template for SCHEMA yielded a collection of highly thermostable CBHII chimeras. Using the computer-generated sequences, a total of 31 new cellulase genes were synthesized and expressed in *Saccharomyces cerevisiae*; each of these cellulases was found to be more stable than the most stable parent cellulase from *H. insolens*, as measured either by half-life of inactivation at 63°C or by *T*_1/2_. These findings demonstrated the value of using structure-guided recombination to discover important sequence-function relationships for efficient generation of highly stable cellulases.

In addition to the improvement of cellulase thermostability, an increase of cellulase stability in detergent solutions following protein engineering has also been reported [58]. *H. insolens *Cel45 endoglucanase is used in the detergent industry, but is inactivated by the detergent C12-LAS (an anionic surfactant) owing to the positive charges of the enzyme surface. Based on the Cel45 crystal structure, different mutations to surface residues were obtained by site-directed mutagenesis. The data on these mutants showed that the introduction of positive charges or removal of negative charges greatly increases detergent sensitivity. The R158E mutation, in particular, gave the highest increase in stability against C12-LAS.

### 5.2. Improvement of Catalytic Activity

The improvement of cellulase catalytic activity using site-directed mutagenesis and directed evolution has attracted considerable attention in recent years. However, owing to the absence of general rules for site-directed mutagenesis and the limitation of screening methods on solid cellulosic substrates for postdirected evolution screening of cellulases with improved activity on insoluble substrates, only a few successful examples of cellulase mutants exist that have significantly higher activity on insoluble substrates [53]. A 20% improvement in the activity of a modified endoglucanase Cel5A from the bacterium *Acidothermus cellulolyticus* has been reported on microcrystalline cellulose following site-directed mutagenesis [59]. A 5-fold higher specific activity in a *Bacillus subtilis* endoglucanase mutant was found following directed evolution [60]. An endocellulase gene from the termite *Reticulitermes speratus* was modified by site-directed mutagenesis, and three mutants, G91A, Y97W, and K429A, displayed higher activities towards carboxymethyl cellulose than the wild type enzyme [61]. Similarly, few reports have been documented thus far on improving the catalytic activity of thermophilic fungal cellulases using either site-directed mutagenesis or directed evolution. As discussed above, the S290T mutant from *M. albomyces* Cel7B exhibits not only improved thermostability but also a 2-fold increase in the rate of Avicel hydrolysis at 70°C [62]. Similar results were also obtained with the *T. emersonii* Cel7A following site-directed mutagenesis [25].

As mentioned previously, and highlighted by recent studies [37], CBDs of cellulases play important roles in enhancing enzymatic activities against crystalline cellulose. A basic approach in CBD engineering is to add or replace a CBD in order to improve hydrolytic activity. Indeed, addition of a CBD from *T. reesei *CBHII to a *T*. *harzianum* chitinase resulted in increased hydrolytic activity on insoluble substrates [63]. The thermophilic fungus *H. grisea* produces two endoglucanases, one with a CBD (EGL3) and one without CBD (EGL4). The fusion protein, EGL4CBD, which consists of the EGL4 catalytic domain and the EGL3 CBD, shows relatively high activity against carboxymethyl cellulose [18]. *M. albomyces *family 7 (Cel7A and Cel7B) and family 45 (Cel45A) glycosyl hydrolases lack a consensus CBD and its associated linker [23]. To improve their efficiency, these three cellulases were genetically modified to carry the CBD of *T. reesei *CBHI. The presence of the CBD was shown to improve their hydrolytic potential towards crystalline cellulose [64].

### 5.3. Conversion to Glycosynthases

An important development in cellulase engineering is the conversion of cellulases to glycosynthases by site-directed mutagenesis [65]. The glycosynthases are retaining glycosidase mutants in which the catalytic nucleophile has been replaced by a non-nucleophilic residue. The first glycosynthase reported from thermophilic fungi was derived from *H. insolens* Cel7B after E197 was mutated to Ala. The resultant Cel7B E197A glycosynthase was able to catalyze the regio- and stereoselective glycosylation of appropriate receptors in high yield [66]. More recently, three mutants of the *H*.* insolens* Cel7B E197A glycosynthase were prepared and characterized by site-directed mutagenesis: E197A/H209A and E197A/H209G double mutants, and the Cel7B E197A/H209A/A211T triple mutant [67]. These second-generation glycosynthase mutants underwent rational redesign in +1 subsite with the aim of broadening the substrate specificity of the glycosynthase. The results showed that the double mutants E197A/H209A and E197A/H209G preferentially catalyze the formation of a *β*-(1,4) linkage between the two disaccharides. In contrast, the single Cel7B mutant E197A and triple Cel7B mutant E197A/H209A/A211T produce predominantly the *β*-(1,3)-linked tetrasaccharide. This work indicated that the regioselectivity of the glycosylation reaction catalyzed by *H. insolens* Cel7B E197A glycosynthase could be modulated by appropriate active-site mutations.

## 6. Conclusions and Future Perspectives

Thermophilic fungal cellulases have recently emerged as promising alternatives in biotechnological applications. However, only a minority of thermophilic fungal cellulases has been characterized in detail so far. Site-directed mutagenesis and directed evolution have been employed and are currently the most preferable approaches to obtain novel thermostable mutants. A systematic characterization of cellulases from additional thermophilic fungi is necessary to better understand their thermostability and evolutionary relationships to mesophilic cellulases. Further improvement of thermophilic fungal celulases will assist in developing better and more versatile cellulases for biotechnological applications and provide novel opportunities in protein engineering efforts.

## Figures and Tables

**Table 1:** Some properties of recombinant thermophilic fungal cellulases expressed in heterologous hosts.

Fungus	Gene	Family	Host	Optimal pH	pI	Optimal Temp (°C)	Thermal stability	Molecular mass (kDa)	Reference
*Acremonium thermophilum*	*cel7a*	7	*Trichoderma reesei*	5.5	4.67	60	NR	53.7	[15]
*Chaetomium thermophilum*	*cel7a*	7	*Trichoderma reesei*	4	5.05	65	NR	54.6	[15]
*Chaetomium thermophilum*	*cbh3*	7	*Pichia pastoris*	4	5.15	60	*T*_1/2_: 45 min at 70°C	50.0	[16]
*Humicola grisea*	*egl2*	5	*Aspergillus oryzae*	5	6.92	75	80% residual activity for 10 min at 75°C	42.6	[17]
*Humicola grisea*	*egl3*	45	*Aspergillus oryzae*	5	5.78	60	75% residual activity for 10 min at 80°C	32.2	[17]
*Humicola grisea*	*egl4*	45	*Aspergillus oryzae*	6	6.44	75	75% residual activity for 10 min at 80°C	24.2	[18]
*Humicola grisea var thermoidea*	*eg1*	7	*Aspergillus oryzae*	5	6.43	55–60	Stable for 10 min at 60°C	47.9	[19]
*Humicola grisea var thermoidea*	*cbh1*	7	*Aspergillus oryzae*	5	4.73	60	Stable for 10 min at 55°C	55.7	[19]
*Humicola insolens*	*avi2*	6	*Humicola insolens*	NR	5.65	NR	NR	51.3	[20]
*Humicola insolens*	*cbhII*	6	*Saccharomyces cerevisiae*	9	NR	57	*T*_1/2_: 95 min at 63°C	NR	[21, 22]
*Melanocarpus albomyces*	*cel7b*	7	*Trichoderma reesei*	6–8	4.23	NR	NR	50.0	[23]
*Melanocarpus albomyces*	*cel7a*	7	*Trichoderma reesei*	6–8	4.15	NR	NR	44.8	[23]
*Melanocarpus albomyces*	*cel45a*	45	*Trichoderma reesei*	6–8	5.22	NR	NR	25.0	[23]
*Talaromyces emersonii*	*cel3a*	3	*Trichoderma reesei*	4.02	3.6	71.5	*T*_1/2_: 62 min at 65°C	90.6	[24]
*Talaromyces emersonii*	*cel7*	7	*E. coli*	5	4.0	68	*T*_1/2_: 68 min at 80°C	48.7	[7]
*Talaromyces emersonii*	*cel7A*	7	*Saccharomyces cerevisiae*	4-5		65	*T*_1/2_: 30 min at 70°C	46.8	[25]
*Thermoascus aurantiacus*	*cbh1*	7	*Saccharomyces cerevisiae*	6	4.37	65	80% residual activity for 60 min at 65°C	48.7	[26]
*Thermoascus aurantiacus*	*eg1*	5	*Saccharomyces cerevisiae*	6	4.36	70	stable for 60 min at 70°C	37.0	[27]
*Thermoascus aurantiacus*	*bgl1*	3	*Pichia pastoris*	5	4.61	70	70% residual activity for 60 min at 60°C	93.5	[28]
*Thermoascus aurantiacus*	*cel7a*	7	*Trichoderma reesei*	5	4.44	65	NR	46.9	[15]

**Table 2:** Thermophilic fungal cellulases with solved 3D structures.

Source	Name	Family	Fold	Reference
*H. insolens*	Cel6A (CBH)	6	*β*/*α*-barrel	[39]
*H. insolens*	Cel6B (EG)	6	*β*/*α*-barrel	[40]
*H. insolens*	EGI	7	*β*-sandwich	[41]
*H. insolens*	Cel7B	7	*β*-sandwich	[42]
*H. insolens*	EGV	45	*β*-barrel	[43]
*H. grisea*	Cel12A	12	*β*-sandwich	[44]
*T. emersonii*	CBHIB	7	*β*-sandwich	[7]
*T. aurantiacus*	Cel5A	5	*β*/*α*-barrel	[45]
*M. albomyces*	maEG	45	*β*-barrel	[46]
*M. albomyces*	Cel7B	7	*β*-sandwich	[47]

## References

[1] Wilson DB (2009). Cellulases and biofuels. *Current Opinion in Biotechnology*.

[2] Sukumaran RK, Surender VJ, Sindhu R (2010). Lignocellulosic ethanol in India: prospects, challenges and feedstock availability. *Bioresource Technology*.

[3] Vlasenko E, Schülein M, Cherry J, Xu F (2010). Substrate specificity of family 5, 6, 7, 9, 12, and 45 endoglucanases. *Bioresource Technology*.

[4] Maheshwari R, Bharadwaj G, Bhat MK (2000). Thermophilic fungi: their physiology and enzymes. *Microbiology and Molecular Biology Reviews*.

[5] Suto M, Tomita F (2001). Induction and catabolite repression mechanisms of cellulase in fungi. *Journal of Bioscience and Bioengineering*.

[6] Murray PG, Collins CM, Grassick A, Tuohy MG (2003). Molecular cloning, transcriptional, and expression analysis of the first cellulase gene (cbh2), encoding cellobiohydrolase II, from the moderately thermophilic fungus Talaromyces emersonii and structure prediction of the gene product. *Biochemical and Biophysical Research Communications*.

[7] Grassick A, Murray PG, Thompson R (2004). Three-dimensional structure of a thermostable native cellobiohydrolase, CBH IB, and molecular characterization of the cel7 gene from the filamentous fungus, Talaromyces emersonii. *European Journal of Biochemistry*.

[8] Pocas-Fonseca MJ, Silva-Pereira I, Rocha BB, Azevedo MDO (2000). Substrate-dependent differential expression of humicola grisea var. thermoidea cellobiohydrolase genes. *Canadian Journal of Microbiology*.

[9] Collins CM, Murray PG, Denman S (2007). Molecular cloning and expression analysis of two distinct *β*-glucosidase genes, bg1 and aven1, with very different biological roles from the thermophilic, saprophytic fungus Talaromyces emersonii. *Mycological Research*.

[10] Benko Z, Drahos E, Szengyel Z, Puranen T, Vehmaanpera J, Reczey K (2007). Thermoascus aurantiacus CBHI/Cel7A production in Trichoderma reesei on alternative carbon sources. *Applied Biochemistry and Biotechnology*.

[11] Ilmen M, Saloheimo A, Onnela ML, Penttila ME (1997). Regulation of cellulase gene expression in the filamentous fungus Trichoderma reesei. *Applied and Environmental Microbiology*.

[12] Furukawa T, Shida Y, Kitagami N (2009). Identification of specific binding sites for XYR1, a transcriptional activator of cellulolytic and xylanolytic genes in Trichoderma reesei. *Fungal Genetics and Biology*.

[13] Soni SK, Soni R (2010). Regulation of cellulase synthesis in Chaetomium erraticum. *BioResources*.

[14] Kumar R, Singh S, Singh OV (2008). Bioconversion of lignocellulosic biomass: biochemical and molecular perspectives. *Journal of Industrial Microbiology and Biotechnology*.

[15] Voutilainen SP, Puranen T, Siika-Aho M (2008). Cloning, expression, and characterization of novel thermostable family 7 cellobiohydrolases. *Biotechnology and Bioengineering*.

[16] Li YL, Li H, Li AN, Li DC (2009). Cloning of a gene encoding thermostable cellobiohydrolase from the thermophilic fungus Chaetomium thermophilum and its expression in Pichia pastoris. *Journal of Applied Microbiology*.

[17] Takashima S, Nakamura A, Hidaka M, Masaki H, Uozumi T (1999). Molecular cloning and expression of the novel fungal *β*-glucosidase genes from Humicola grisea and Trichoderma reesei. *Journal of Biochemistry*.

[18] Takashima S, Iikura H, Nakamura A, Hidaka M, Masaki H, Uozumi T (1999). Comparison of gene structures and enzymatic properties between two endoglucanases from Humicola grisea. *Journal of Biotechnology*.

[19] Takashima S, Nakamura A, Hidaka M, Masaki H, Uozumi T (1996). Cloning, sequencing, and expression of the cellulase genes of Humicola grisea var. thermoidea. *Journal of Biotechnology*.

[20] Moriya T, Watanabe M, Sumida N, Okakura K, Murakami T (2003). Cloning and overexpression of the avi2 gene encoding a major cellulase produced by Humicola insolens FERM BP-5977. *Bioscience, Biotechnology and Biochemistry*.

[21] Heinzelman P, Snow CD, Wu I (2009). A family of thermostable fungal cellulases created by structure-guided recombination. *Proceedings of the National Academy of Sciences of the United States of America*.

[22] Heinzelman P, Snow CD, Smith MA (2009). SCHEMA recombination of a fungal cellulase uncovers a single mutation that contributes markedly to stability. *Journal of Biological Chemistry*.

[23] Haakana H, Miettinen-Oinonen A, Joutsjoki V, Mantyla A, Suominen P, Vehmaanperä J (2004). Cloning of cellulase genes from Melanocarpus albomyces and their efficient expression in Trichoderma reesei. *Enzyme and Microbial Technology*.

[24] Murray P, Aro N, Collins C (2004). Expression in Trichoderma reesei and characterisation of a thermostable family 3 *β*-glucosidase from the moderately thermophilic fungus Talaromyces emersonii. *Protein Expression and Purification*.

[25] Voutilainen SP, Murray PG, Tuohy MG, Koivula A (2010). Expression of Talaromyces emersonii cellobiohydrolase Cel7A in Saccharomyces cerevisiae and rational mutagenesis to improve its thermostability and activity. *Protein Engineering, Design and Selection*.

[26] Hong J, Tamaki H, Yamamoto K, Kumagai H (2003). Cloning of a gene encoding thermostable cellobiohydrolase from Thermoascus aurantiacus and its expression in yeast. *Applied Microbiology and Biotechnology*.

[27] Hong J, Tamaki H, Yamamoto K, Kumagai H (2003). Cloning of a gene encoding a thermo-stable endo-*β*-1,4-glucanase from Thermoascus aurantiacus and its expression in yeast. *Biotechnology Letters*.

[28] Hong J, Tamaki H, Kumagai H (2007). Cloning and functional expression of thermostable *β*-glucosidase gene from Thermoascus aurantiacus. *Applied Microbiology and Biotechnology*.

[29] Jeoh T, Michener W, Himmel ME, Decker SR, Adney WS (2008). Implications of cellobiohydrolase glycosylation for use in biomass conversion. *Biotechnol Biofuels*.

[30] Meldgaard M, Svendsen I (1994). Different effects of N-glycosylation on the thermostability of highly homologous bacterial (1, 3-1, 4)-*β*-glucanases secreted from yeast. *Microbiology*.

[31] Mamma D, Hatzinikolaou DG, Christakopoulos P (2004). Biochemical and catalytic properties of two intracellular *β*-glucosidases from the fungus Penicillium decumbens active on flavonoid glucosides. *Journal of Molecular Catalysis B*.

[32] Pack SP, Yoo YJ (2004). Protein thermostability: structure-based difference of amino acid between thermophilic and mesophilic proteins. *Journal of Biotechnology*.

[33] Trivedi S, Gehlot HS, Rao SR (2006). Protein thermostability in Archaea and Eubacteria. *Genetics and Molecular Research*.

[34] Taylor TJ, Vaisman II (2010). Discrimination of thermophilic and mesophilic proteins. *BMC Structural Biology*.

[35] Beckham GT, Bomble YJ, Matthews JF (2010). The O-glycosylated linker from the Trichoderma reesei family 7 cellulase is a flexible, disordered protein. *Biophysical Journal*.

[36] Hashimoto H (2006). Recent structural studies of carbohydrate-binding modules. *Cellular and Molecular Life Sciences*.

[37] Shoseyov O, Shani Z, Levy I (2006). Carbohydrate binding modules: biochemical properties and novel applications. *Microbiology and Molecular Biology Reviews*.

[38] Dagel DJ, Liu YS, Zhong L (2011). In situ imaging of single carbohydrate-binding modules on cellulose microfibrils. *Journal of Physical Chemistry B*.

[39] Varrot A, Frandsen TP, von Ossowski I (2003). Structural basis for ligand binding and processivity in cellobiohydrolase Cel6A from Humicola insolens. *Structure*.

[40] Davies GJ, Brzozowski AM, Dauter M, Varrot A, Schulein M (2000). Structure and function of Humicola insolens family 6 cellulases: structure of the endoglucanase, Cel6B, at 1.6 Å resolution. *Biochemical Journal*.

[41] Davies GJ, Ducros V, Lewis RJ, Borchert TV, Schulein M (1997). Oligosaccharide specificity of a family 7 endoglucanase: insertion of potential sugar-binding subsites. *Journal of Biotechnology*.

[42] Mackenzie LF, Sulzenbacher G, Divne C (1998). Crystal structure of the family 7 endoglucanase I (Cel7B) from Humicola insolens at 2.2 Å resolution and identification of the catalytic nucleophile by trapping of the covalent glycosyl-enzyme intermediate. *Biochemical Journal*.

[43] Davies GJ, Dodson GG, Hubbard RE (1993). Structure and function of endoglucanase V. *Nature*.

[44] Sandgren M, Berglund GI, Shaw A (2004). Crystal complex structures reveal how substrate is bound in the -4 to the +2 binding sites of Humicola grisea Cel12A. *Journal of Molecular Biology*.

[45] Lo Leggio L, Larsen S (2002). The 1.62 Å structure of Thermoascus aurantiacus endoglucanase: completing the structural picture of subfamilies in glycoside hydrolase family 5. *FEBS Letters*.

[46] Hirvonen M, Papageorgiou AC (2003). Crystal structure of a family 45 endoglucanase from Melanocarpus albomyces: mechanistic implications based on the free and cellobiose-bound forms. *Journal of Molecular Biology*.

[47] Parkkinen T, Koivula A, Vehmaanpera J, Rouvinen J (2008). Crystal structures of Melanocarpus albomyces cellobiohydrolase Cel7B in complex with cello-oligomers show high flexibility in the substrate binding. *Protein Science*.

[48] Takashima S, Iikura H, Nakamura A, Hidaka M, Masaki H, Uozumi T (1998). Isolation of the gene and characterization of the enzymatic properties of a major exoglucanase of Humicola grisea without a cellulose-binding domain. *Journal of Biochemistry*.

[49] Takashima S, Ohno M, Hidaka M, Nakamura A, Masaki H, Uozumi T (2007). Correlation between cellulose binding and activity of cellulose-binding domain mutants of Humicola grisea cellobiohydrolase 1. *FEBS Letters*.

[50] Potterton L, McNicholas S, Krissinel E (2004). Developments in the CCP4 molecular-graphics project. *Acta Crystallographica Section D*.

[51] Sandgren M, Gualfetti PJ, Paech C (2003). The Humicola grisea Cell2A enzyme structure at 1.2 Å resolution and the impact of its free cysteine residues on thermal stability. *Protein Science*.

[52] Valjakka J, Rouvinen J (2003). Structure of 20K endoglucanase from Melanocarpus albomyces at 1.8 Å resolution. *Acta Crystallographica D*.

[53] Percival Zhang YH, Himmel ME, Mielenz JR (2006). Outlook for cellulase improvement: screening and selection strategies. *Biotechnology Advances*.

[54] Labrou NE (2010). Random mutagenesis methods for in vitro directed enzyme evolution. *Current Protein and Peptide Science*.

[55] Voutilainen SP, Boer H, Linder MB (2007). Heterologous expression of *Melanocarpus albomyces* cellobiohydrolase Cel7B, and random mutagenesis to improve its thermostability. *Enzyme and Microbial Technology*.

[56] Sandgren M, Stahlberg J, Mitchinson C (2005). Structural and biochemical studies of GH family 12 cellulases: improved thermal stability, and ligand complexes. *Progress in Biophysics and Molecular Biology*.

[57] Yennamalli RM, Rader AJ, Wolt JD, Sen TZ (2011). Thermostability in endoglucanases is fold-specific. *BMC Structural Biology*.

[58] Otzen DE, Christiansen L, Schulein M (1999). A comparative study of the unfolding of the endoglucanase Ce145 from Humicola insolens in denaturant and surfactant. *Protein Science*.

[59] Mccarter SL, Adney WS, Vinzant TB (2002). Exploration of cellulose surface-binding properties of Acidothermus cellulolyticus Cel5A by site-specific mutagenesis. *Applied Biochemistry and Biotechnology A*.

[60] Kim YS, Jung HC, Pan JG (2000). Bacterial cell surface display of an enzyme library for selective screening of improved cellulase variants. *Applied and Environmental Microbiology*.

[61] Ni J, Takehara M, Watanabe H (2010). Identification of activity related amino acid mutations of a GH9 termite cellulase. *Bioresource Technology*.

[62] Voutilainen SP, Boer H, Alapuranen M, Janis J, Vehmaanpera J, Koivula A (2009). Improving the thermostability and activity of Melanocarpus albomyces cellobiohydrolase Cel7B. *Applied Microbiology and Biotechnology*.

[63] Limon MC, Margolles-Clark E, Benitez T, Penttila M (2001). Addition of substrate-binding domains increases substrate-binding capacity and specific activity of a chitinase from Trichoderma harzianum. *FEMS Microbiology Letters*.

[64] Szijarto N, Siika-aho M, Tenkanen M (2008). Hydrolysis of amorphous and crystalline cellulose by heterologously produced cellulases of Melanocarpus albomyces. *Journal of Biotechnology*.

[65] Shaikh FA, Withers SG (2008). Teaching old enzymes new tricks: engineering and evolution of glycosidases and glycosyl transferases for improved glycoside synthesis. *Biochemistry and Cell Biology*.

[66] Fort S, Boyer V, Greffe L (2000). Highly efficient synthesis of *β*(1 − >4)-oligo- and -polysaccharides using a mutant cellulase. *Journal of the American Chemical Society*.

[67] Blanchard S, Armand S, Couthino P (2007). Unexpected regioselectivity of Humicola insolens Cel7B glycosynthase mutants. *Carbohydrate Research*.

